# Stationäre und intensivmedizinische Versorgungsstrukturen von COVID-19-Patienten bis Juli 2020

**DOI:** 10.1007/s00063-021-00776-6

**Published:** 2021-01-26

**Authors:** C. Hentschker, C. Mostert, J. Klauber, J. Malzahn, D. Scheller-Kreinsen, G. Schillinger, C. Karagiannidis, R. Busse

**Affiliations:** 1 Wissenschaftliches Institut der AOK (WIdO), Berlin, Deutschland; 2 AOK-Bundesverband, Berlin, Deutschland; 3 ARDS/ECMO-Zentrum Köln-Merheim, Kliniken der Stadt Köln, Universität Witten/Herdecke, Witten, Deutschland; 4 Fachgebiet Management im Gesundheitswesen, Technische Universität Berlin, Str. des 17. Juni 135, 10623 Berlin, Deutschland

**Keywords:** Vorerfahrung, Patientensteuerung, Stufenkonzept, Patientenverteilung, Administrative Daten, Previous experience, Patient management, Pyramid-type concept, Distribution of patients, Administrative date

## Abstract

**Hintergrund:**

Hospitalisierte COVID-19-Patienten weisen eine hohe Morbidität und Mortalität auf und sind häufig auf eine intensivstationäre Behandlung und hier vor allem auf eine Beatmungstherapie angewiesen. Bisher ist wenig über die Patientenallokation bekannt.

**Ziel der Arbeit:**

Die Darstellung der Strukturen der Krankenhausversorgung der COVID-19-Patienten zwischen dem 26. Februar bis zum 31. Juli 2020

**Daten und Methoden:**

Für die Analyse der Versorgungsstrukturen wurden die Abrechnungsdaten der Allgemeinen Ortskrankenkassen (AOK) ausgewertet. Es wurden ausschließlich abgeschlossene somatische COVID-19-Fälle ausgewertet, bei denen das Virus durch einen Labortest nachgewiesen wurde. Die Stichprobe umfasst 17.094 COVID-19-Fälle, deren Behandlung in 1082 Krankenhäusern erfolgte.

**Ergebnisse:**

An der Versorgung der COVID-19-Fälle waren 77 % aller Krankenhäuser beteiligt, an der intensivmedizinischen Behandlung 48 % aller Krankenhäuser. Von den Krankenhäusern, die COVID-19-Fälle behandelt haben, versorgte eine Hälfte 88 % aller Fälle. Das deutet zwar auf einen Zentrierungseffekt der COVID-19-Fälle auf bestimmte Krankenhäuser hin, jedoch verteilten sich die übrigen 12 % der Fälle auf viele Krankenhäuser mit oftmals sehr kleinen Fallzahlen. Des Weiteren wurde knapp ein Viertel der beatmeten COVID-19-Fälle in Krankenhäusern behandelt, die eine unterdurchschnittliche Beatmungserfahrung aufweisen.

**Diskussion:**

Im Rahmen steigender Infektionszahlen ist es sowohl notwendig die Versorgungsstrukturen von COVID-19-Fällen durch klar definierte und zentral gesteuerte Stufenkonzepte zu verbessern als auch die Versorgung der Patienten ohne COVID-19 weiterhin aufrechtzuerhalten. Ein umfassendes Stufenkonzept mit stärkerer Konzentration erscheint für die Versorgung dieser komplex erkrankten Patienten sinnvoll.

**Zusatzmaterial online:**

Die Onlineversion dieses Beitrags (10.1007/s00063-021-00776-6) enthält die Tabelle S1 und die Abbildungen S1 bis S3. Beitrag und Zusatzmaterial stehen Ihnen auf www.springermedizin.de zur Verfügung. Bitte geben Sie dort den Beitragstitel in die Suche ein, das Zusatzmaterial finden Sie beim Beitrag unter „Ergänzende Inhalte“.

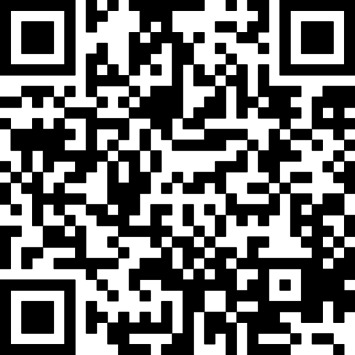

## Abstract (en)

**Background:**

Hospitalized coronavirus disease 2019 (COVID-19) patients have a high morbidity and mortality and are often dependent on intensive care, especially mechanical ventilation. Little is as yet known about COVID-19 patient allocation.

**Objectives:**

Analysis of the structures of German hospital care for COVID-19 patients up to July 2020 in terms of number of beds and previous ventilation experience.

**Data and methods:**

For the analysis of the care structures, only completed COVID-19 cases in which the virus was detected by a PCR test were evaluated. Claims data from the German Local Health Care Funds (Allgemeine Ortskrankenkassen, AOK) were analysed. The sample includes 17,094 COVID-19 cases that were treated in 1082 hospitals.

**Results:**

A total of 77% of all hospitals participated in the treatment COVID-19 patients and 48% of all hospitals provided intensive care for these patients. One half of the hospitals that treated COVID-19 cases cared for 88% of all cases. Although this suggests a centralization effect of COVID-19 cases in specific hospitals, the remaining 12% of the cases were distributed among many hospitals with often very small numbers of cases. Furthermore, 23% of the ventilated COVID-19 cases were treated in hospitals with below-average ventilation experience.

**Conclusions:**

In the context of increasing numbers of infections, it is both necessary to improve the allocation of hospitalized, and therefore potentially ventilated, COVID-19 cases by means of clearly defined and centrally controlled pyramid-type concepts and to continue to care for patients without COVID-19. For Germany, a comprehensive pyramid-type concept with a greater concentration in the best-qualified hospitals seems reasonable for the care of these patients with complex diseases.

## Einleitung

Deutschlandweit haben sich bisher (Stand: 19.01.2021) mehr als 2 Millionen Menschen mit dem Coronavirus (SARS-CoV-2) infiziert, von denen bisher mehr als 47.000 verstorben sind. Patienten, die aufgrund von COVID-19 im Krankenhaus behandelt wurden, sind im Median über 70 Jahre alt, weisen in der Regel Begleiterkrankungen auf und jeder 6. Patient muss beatmet werden [7]. Etwa 22 % der stationär behandelten Patienten verstarben im Krankenhaus, bei den beatmeten Patienten sogar mehr als die Hälfte. Aus pathophysiologischer Sicht ist COVID-19 eine neuartige Erkrankung, die verschiedene Organsysteme betrifft und eine hohe Fachexpertise bei der Behandlung erfordert [1, 8]. Generell stellen Pandemien Krankenhäuser vor besondere Herausforderungen [11]. Die Meldungen über die stark ansteigenden Krankenhausfallzahlen in Europa, beispielsweise in Frankreich oder in Italien, haben dazu geführt, dass in Deutschland schon frühzeitig Anreize geschaffen wurden, um die Zahl der Intensivbetten und Beatmungsgeräte zu steigern. Im Rahmen der Notfallmaßnahmen zur Eindämmung und Beherrschung der Pandemie waren Krankenhäuser dazu angehalten, alle planbaren Eingriffe und Operationen – soweit medizinisch vertretbar – zu verschieben, um Kapazitäten für die Behandlung von COVID-19-Erkrankten freizuhalten [3]. Zusammen mit der zurückgegangenen Nachfrage nach stationären Leistungen bei den Patienten kam es zu einem starken Rückgang der Krankenhausfallzahlen [2, 4].

Vor diesem Hintergrund ist es wichtig, die Strukturen der Krankenhausversorgung der COVID-19-Patienten näher zu betrachten und zu untersuchen, wie viele bzw. welche Krankenhäuser und in welchem Umfang an der Versorgung von COVID-19-Patienten beteiligt waren. Insbesondere die Beatmungstherapie stellt Intensivmediziner vor tagtägliche Herausforderungen im Umgang mit den Patienten aufgrund des langen Verlaufs und der vielfältigen Komplikationen. Diese Arbeit soll die Patientenallokation innerhalb der ersten Welle beschreiben und eine Grundlage für eine Diskussion bieten, ob COVID-19-Patienten schwerpunktmäßig an Krankenhäusern mit einer bestimmten Ausstattung und Erfahrung behandelt werden sollten.

## Daten und Methodik

Die vorliegenden Auswertungen basieren auf den Abrechnungsdaten der Allgemeinen Ortskrankenkassen (AOK) (§ 301 SGB V). In diesen sind alle Krankenhausaufenthalte der AOK-Versicherten enthalten. Die Daten umfassen Informationen zum Behandlungsfall wie Alter, Geschlecht, Zeitraum der Behandlung, Haupt- und Nebendiagnosen sowie die durchgeführten Operationen und Prozeduren. Krankenhaustandorte, die aufgrund von Verbundbildungen unter einem Institutionskennzeichen abrechnen, sind in den Daten nicht zu unterscheiden und werden im Folgenden als ein Krankenhaus betrachtet.

Für die Analyse der Versorgungsstrukturen wurden ausschließlich abgeschlossene somatische COVID-19 -Fälle ausgewertet, bei denen das Virus durch einen Labortest nachgewiesen wurde (Diagnose-Code: U07.1!). Eingeschlossen wurden Patienten, die zwischen dem 26.02.2020 und dem 31.07.2020 aufgenommen wurden und mindestens 18 Jahre alt waren. Im Behandlungsverlauf kann es zu Verlegungen von Patienten zu einem anderen Krankenhaus kommen. Um die Versorgungsstrukturen der COVID-19-Patienten abzubilden, wurden alle Fälle der verlegten Patienten getrennt betrachtet, da das Gesamtversorgungsgeschehen abgebildet werden soll.

Um die Grundgesamtheit der Krankenhäuser in den Daten zu ermitteln, wurden Krankenhäuser betrachtet, die im Jahr 2019 und 2020 mindestens einen somatischen AOK-Fall abgerechnet haben und ein gültiges Institutionskennzeichen aufweisen. Neben reinen psychiatrischen und psychosomatischen Krankenhäusern wurden reine Kinderkliniken aus der Grundgesamtheit ausgeschlossen, d. h.: Der Anteil von Patienten im Alter von 18 Jahren oder älter musste mindestens 20 % betragen. Insgesamt wurden 1412 Krankenhäuser in die Analysen einbezogen.

## Ergebnisse

Im Zeitraum zwischen dem 26.02.2020 und dem 31.07.2020 wurden insgesamt 15.166 AOK-Patienten mit COVID-19 stationär behandelt; davon wurden 2299 Patienten (15,2 %) beatmet. Das Durchschnittsalter betrug 67 Jahre. Bei 1631 Patienten (10,8 %) kam es zu Verlegungen zwischen Krankenhäusern. Bei den beatmeten Patienten waren es 31,9 % (734/2299), bei den Patienten ohne Beatmung 7,0 % (897/12.867). Somit umfassen die 15.166 AOK-Patienten 17.094 Fälle, deren Behandlung in 1082 Krankenhäusern erfolgte. Da insgesamt 1412 Krankenhäuser stationäre Krankenhausfälle mit der AOK abgerechnet haben, waren 23 % (330/1412) der Krankenhäuser nicht an der Versorgung von COVID-19-Fällen beteiligt.

### COVID-19-Fallzahlen pro Krankenhaus

**Figure 1:**
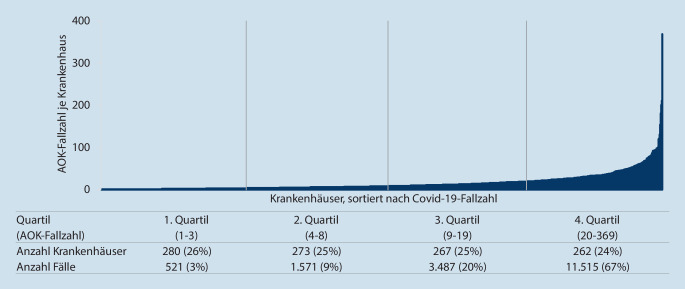

In Abb. 1 wird die Verteilung der Krankenhäuser, die COVID-19-Patienten behandelt haben, aufsteigend sortiert nach ihrer COVID-19-Fallzahl (AOK-Fälle) gezeigt. Auf Basis der Fallzahl wurden die Krankenhäuser in Quartile eingeteilt. Im Quartil mit der niedrigsten Fallzahl haben die Krankenhäuser 1–3 AOK-COVID-19-Fälle behandelt. Insgesamt behandelten hier 26 % (*n* = 280) der Krankenhäuser rund 3 % (*n* = 521) der Fälle. Dahingegen behandelten im Quartil mit den höchsten Fallzahlen 24 % (*n* = 262) der Krankenhäuser 67 % (*n* = 11.515) der Fälle.

**Figure 2:**
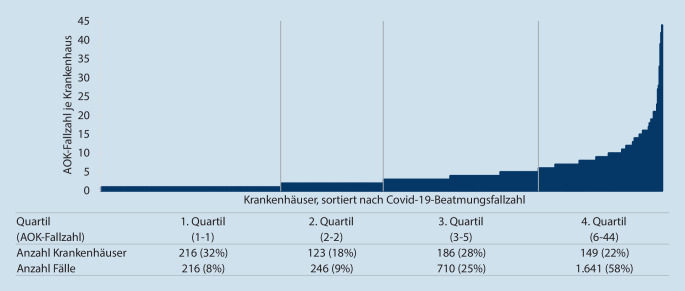

Bei den beatmeten COVID-19-Fällen ist eine ähnliche Verteilung zu beobachten (Abb. 2). So behandelten beispielsweise 22 % (*n* = 149) der Krankenhäuser im Quartil mit den höchsten Fallzahlen 58 % (*n* = 1641) der Fälle. Insgesamt wurden AOK-versicherte COVID-19-Patienten in 674 Krankenhäuser beatmet, d. h. in 62 % (674/1082) der Krankenhäuser mit stationären COVID-19-Patienten und in 48 % (674/1412) aller Krankenhäuser.

### Fallzahlen nach Krankenhausstrukturen

Krankenhäuser haben eine unterschiedliche Vorerfahrung bzw. Ausstattung im Umgang mit beatmeten und hochinfektiösen Patienten. Schätzparameter hierfür sind die Bettenzahl des Krankenhauses und die Summe der Beatmungsstunden bei Fällen mit einer Pneumonie, Sepsis oder Atemnotsyndrom (ARDS) als Haupt- oder Nebendiagnose im Jahr 2019, da dies die führenden Diagnosen bei komplex erkrankten Beatmungspatienten sind. Insbesondere diese Diagnosen kommen dem Verlauf der COVID-19-Erkrankung am nächsten. Die Bettenzahl ist ein verhältnismäßig unspezifisches Maß, das jedoch Hinweise auf die Ausstattung des Krankenhauses geben kann. So weisen Krankenhäuser mit mehr Betten in der Regel mehr Fachabteilungen, eine bessere Ausstattung mit technischen Geräten sowie mehr spezialisiertes Fachpersonal auf. Die Beatmungsstunden bei Fällen mit Pneumonie, Sepsis oder ARDS im Vorjahr stehen näherungsweise für die potenzielle Vorerfahrung des Krankenhauses mit beatmeten Patienten. Die Krankenhäuser werden hierfür in Quartile eingeteilt. Die Krankenhäuser im 1. Quartil entsprechen beispielsweise den 25 % der Krankenhäuser, die die wenigsten Beatmungsstunden für AOK-versicherte Patienten im Jahr 2019 aufgewiesen haben. Zu besseren Einordnung sei erwähnt, dass (i) ein über ein Jahr durchgehend genutzter Beatmungsplatz 8760 h entspräche und (ii) die durchschnittliche Beatmungsdauer der COVID-19-Fälle bei 12,3 Tagen, d. h. bei ca. 300 h, liegt.

**Figure 3:**
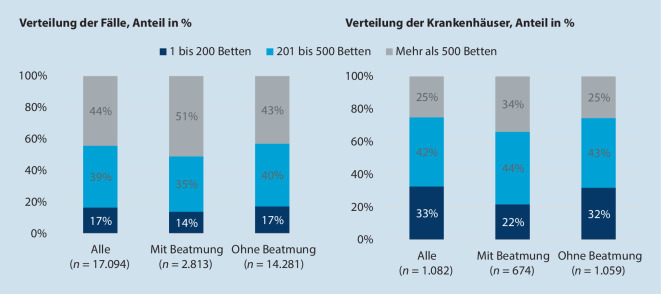

Die Verteilung der COVID-19-Fälle und Krankenhäuser nach der Bettengröße des behandelnden Krankenhauses wird in Abb. 3 gezeigt. Es werden 51 % (1432/2813) der beatmeten Fälle und 43 % (6117/14.281) der Fälle ohne Beatmung in Krankenhäusern mit mehr als 500 Betten behandelt. Hingegen werden 14 % (389/2813) der beatmeten Fälle und 17 % (2453/14.281) der Fälle ohne Beatmung in Krankenhäusern mit nur bis zu 200 Betten behandelt.

Insgesamt haben 33 % (353/1082) der Krankenhäuser, die COVID-19-Patienten behandeln, nur bis zu 200 Betten. Bei den Krankenhäusern, die beatmete Patienten behandeln, liegt deren Anteil mit 22 % (147/674) niedriger (siehe auch Tabelle S1 im elektronischen Zusatzmaterial).

**Figure 4:**
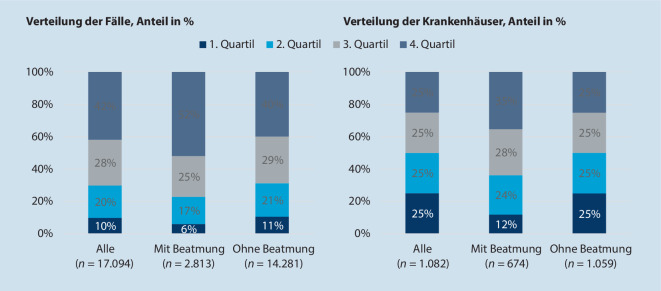

Es wurden 52 % (1461/2813) der beatmeten COVID-19-Fälle in Krankenhäusern behandelt, die im Jahr 2019, gemessen an den Beatmungsstunden bei Fällen mit Pneumonie, Sepsis und ARDS, die meiste Vorerfahrung (4. Quartil) aufwiesen (Abb. 4). Allerdings wurden auch 23 % (643/2813, 1. und 2. Quartil) der beatmeten Fälle in den 36 % (244/674) der Krankenhäuser behandelt, die im Jahr 2019 eine deutlich geringere Beatmungsstundenanzahl aufwiesen. Die Beatmungsstunden bei AOK-Versicherten lagen hier bei nur bis zu 6622 h, verglichen mit der Beatmungsstundenanzahl von Krankenhäusern im 4. Quartil von mehr als 14.052 h weniger als halb so hoch. In diesen Krankenhäusern war die Wegverlegungsrate von COVID-19-Fällen mehr als doppelt so hoch (30 %, 194/643) als bei den Krankenhäusern im 4. Quartil (13 %, 186/1461). Die 25 % (264/1059) der Krankenhäuser mit der geringsten Beatmungserfahrung, die COVID-19-Fälle ohne Beatmung behandeln, weisen nur bis zu 2797 Beatmungsstunden im Jahr 2019 auf. Insgesamt weisen 81 % (287/353) der Krankenhäuser mit bis zu 200 Betten eine geringe Beatmungserfahrung auf, d. h. liegen im 1. oder 2. Quartil bei den Beatmungsstunden.

Abbildung S1 im elektronischen Zusatzmaterial zeigt die Verteilung der COVID-19-Fälle und Krankenhäuser nach den Beatmungsstunden der Krankenhäuser im Jahr 2019 und nach Aufnahmemonat. Es ist ersichtlich, dass der Anteil der Patienten, die in Krankenhäusern mit der meisten Beatmungserfahrung behandelt wurden, relativ konstant zwischen 41–45 % für die Monate März bis Juli lag. Nur im Juni lag der Anteil mit 51 % etwas höher. Der Anteil der Krankenhäuser mit der meisten Beatmungserfahrung stieg zwar leicht an (35 % im Juli gegenüber 28 % im März), dies hatte aber keine Auswirkung auf die Verteilung der Patienten.

### Krankenhäuser ohne COVID-19-Fälle

Bisher wurden nur Krankenhäuser betrachtet, die an der Versorgung von COVID-19-Patienten beteiligt waren. Ein weiterer Aspekt ist, wie sich diese Krankenhäuser auf die Grundgesamtheit aller Krankenhäuser verteilen. Insgesamt haben 77 % (1082/1412) der Krankenhäuser mindestens einen AOK-COVID-19-Fall versorgt. Fast alle Krankenhäuser mit mehr als 500 Betten (98 %, 271/277) waren an der COVID-19-Versorgung beteiligt (Abbildung S2 im elektronischen Zusatzmaterial). Bei den Krankenhäusern zwischen 201 und 500 Betten waren es noch 89 % (458/516). Bei den kleinen Krankenhäusern mit bis zu 200 Betten waren es nur 57 % (353/619). Bei den kleinen Krankenhäusern, die nicht an der Versorgung beteiligt waren, handelt es sich in der Regel um Fachkliniken, die für die Versorgung von COVID-19-Patienten auch nicht infrage kommen. Bei der Verteilung nach Beatmungsstunden ergibt sich ein ähnliches Bild wie bei der Verteilung nach Betten. So waren 97 % der Krankenhäuser mit der meisten Beatmungserfahrung an der COVID-19-Versorgung beteiligt. Bei den Krankenhäusern mit der geringsten Beatmungserfahrung waren es nur 50 %.

### Analyse der Verlegungen

**Figure 5:**
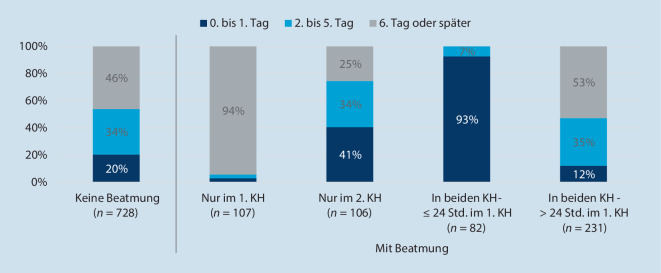

Von den 1631 (10,8 % von 15.166) verlegten COVID-19-Patienten wurden 1261 (77 %) einmalig verlegt, d. h. in je 2 Krankenhäusern behandelt. Bei diesen Verlegungen unterschieden werden COVID-19-Patienten ohne Beatmung sowie Patienten, die nur im ersten Krankenhaus, nur im zweiten Krankenhaus oder in beiden behandelnden Krankenhäusern beatmet worden sind^1^. Entscheidend für eine Verlegung sind der Zeitpunkt der Verlegung sowie die Art der Verlegung. Die Art der Verlegung bestimmt sich aus dem Anteil der Patienten, die in Krankenhäuser verlegt worden sind, die mehr Beatmungsstunden bei Fällen mit Pneumonie, Sepsis und ARDS im Jahr 2019 aufweisen als die erstbehandelnden wegverlegenden Krankenhäuser, und spiegelt den Anteil der Patienten wider, die in ein potenziell erfahreneres Krankenhaus weiterverlegt worden sind. Abb. 5 und Abbildung S3 im elektronischen Zusatzmaterial beschreiben die Gruppe der verlegten Patienten nach dem Zeitpunkt der Verlegung und nach der Art der Verlegung.

Bei den verlegten Patienten ohne Beatmung wurden 20 % (148/728) bereits am Aufnahme- oder am Folgetag verlegt. Bei 50 % (365/728) erfolgte die Verlegung in ein erfahreneres Krankenhaus gemessen an den Beatmungsstunden des Vorjahrs. Bei 94 % (101/107) der Patienten, die ausschließlich im ersten Krankenhaus beatmet wurden, wurden am 6. Tag oder später verlegt. Die Verlegung erfolgte bei 71 % (76/107) der Patienten in ein Krankenhaus mit weniger Beatmungserfahrung als das Krankenhaus, das die Erstbehandlung durchgeführt hat.

Bei den Patienten, die ausschließlich im Krankenhaus nach der Verlegung beatmet wurden, verhält es sich genau umgekehrt. Es wurden 80 % (85/106) dieser Patienten in ein Krankenhaus mit mehr Beatmungserfahrung verlegt. Die Verlegung erfolgte für 41 % (43/106) der Patienten direkt am Aufnahme- oder am Folgetag. Definitionsbedingt wurden 93 % (76/82) der Patienten, die nur 24 h im ersten Krankenhaus beatmet wurden, am Aufnahmetag oder Tag nach der Aufnahme verlegt. Die Verlegung erfolgte meistens in ein erfahreneres Krankenhaus (90 %, 77/86).

Patienten, die in beiden Krankenhäusern beatmet wurden und davon im ersten Krankenhaus länger als 24 h, wurden eher spät verlegt. Bei 35 % dieser Patienten geschah dies zwischen dem 2. und 5. Tag, bei 53 % (122/231) erst am oder nach dem 6. Tag nach Beatmungsbeginn. Die Mehrheit dieser Patienten (77 %, 181/234) wurde in ein erfahreneres Krankenhaus verlegt.

## Diskussion

Die vorliegende Arbeit stellt erstmalig die Verteilung der COVID-19-Patienten im Rahmen der ersten Erkrankungswelle auf die Krankenhäuser in Deutschland dar und zeigt den Grad der Zentrierung der Versorgung auf. Die Analysen zeigen, dass insgesamt über drei Viertel der Krankenhäuser (77 %, 1082/1412) an der Versorgung von COVID-19-Patienten beteiligt waren. Bei der Behandlung beatmeter Fälle waren es 48 % (674/1412) aller Krankenhäuser. Bei den Krankenhäusern mit COVID-19-Fällen hatten viele jedoch nur eine sehr niedrige Fallzahl: So versorgte die Hälfte der beteiligten Häuser (*n* = 553) nur 12 % (2092/17.094) der Patienten (Abb. 1). Die andere Hälfte der Einrichtungen (*n* = 529) versorgte hingegen 88 % (15.002/17.094) der Fälle. Auch bei den Krankenhäusern, die Beatmungen durchführten, beatmeten die fallzahlstärksten 335 Krankenhäuser bereits 83 % (2351/2813) der COVID-19-Fälle. Diese Zahlen deuten zwar auf einen Zentrierungseffekt der COVID-19-Fälle auf bestimmte Krankenhäuser hin, jedoch verteilen sich die übrigen Fälle auf viele Krankenhäuser mit oftmals sehr kleinen Fallzahlen. Bei der Interpretation der Fallzahlen je Einrichtung ist zu berücksichtigen, dass in der ersten Welle die regionale Belastung insgesamt sehr unterschiedlich war.

Dass es im Hinblick auf die Versorgungssteuerung Verbesserungspotenzial gibt, verdeutlichen auch die Ergebnisse zur Verteilung der COVID-19-Patienten auf die Krankenhäuser mit viel und mit wenig Vorerfahrung. Insgesamt wurde knapp ein Viertel der beatmeten COVID-19-Fälle in 244 Krankenhäuser behandelt, die mit nur bis zu rund 6600 Beatmungsstunden im Vorjahr bei AOK-versicherten Patienten mit Pneumonie, Sepsis und ARDS deutlich weniger Vorerfahrung mit (Langzeit‑)Beatmungen haben dürften, als die restlichen an der Beatmung von COVID-19-Fällen beteiligten Krankenhäuser. Von den nichtbeatmungspflichtigen Fällen wurde ein Drittel in Krankenhäusern mit dieser verhältnismäßig geringeren Vorerfahrung behandelt (Abb. 4). Zwar wird diese spezifische Erfahrung für viele dieser Patienten zunächst nicht gebraucht, von Nachteil könnte es aber sein, wenn im Behandlungsverlauf Komplikationen auftreten bzw. eine Beatmung erforderlich wird. Ferner wurde insgesamt über die Hälfte der Fälle in eher kleineren Krankenhäusern mit maximal 500 Betten versorgt (Abb. 3). Anhand der Bettenzahl kann zwar nicht pauschal geschlussfolgert werden, dass die kleineren Krankenhäuser in keinem Fall genauso gut geeignet sind wie größere, aber kleinere Krankenhäuser weisen in der Regel weniger Beatmungserfahrung auf. Des Weiteren kann die bei schwierigen Verläufen erforderliche Vorhaltung unterschiedlicher Fachdisziplinen infrage gestellt werden. Dafür, dass kleinere Krankenhäuser in der Regel als weniger geeignet als andere Krankenhäuser für die Versorgung von COVID-19-Patienten eingeschätzt wurden, spricht die geringe Teilhabe an der Versorgung bzw. deren höhere Wegverlegungsrate.

Insgesamt waren fast alle Krankenhäuser mit Vorerfahrung bzw. vielen Betten an der Versorgung beteiligt (Abb. S2). Da diese Krankenhäuser insbesondere in Ballungsräumen zum Teil nahe beieinanderliegen, steht auch hier die Forderung nach einer besseren Zentralisierung im Raum. Zum einen gelingt bei neuen Krankheitsbildern der Erkenntnisgewinn mit größeren Patientenzahlen pro Haus schneller und der Erfahrungsaustausch unter den Klinikern durch Konzentration wird vereinfacht. Zum anderen ist zu beachten, dass nicht nur die Versorgung der COVID-19-Patienten bestmöglich erfolgt, sondern während der Pandemie auch die Versorgung aller weiteren Krankenhauspatienten aufrechterhalten werden sollte. Klare planbasierte Separierungen von Versorgungsaufgaben in Verdichtungsgebieten stationärer Versorgungsangebote tragen zur Aufrechterhaltung einer qualitativ hochwertigen Gesamtversorgung bei.

COVID-19 ist eine Erkrankung, die viele Organsysteme und damit auch Fachdisziplinen betreffen kann und somit eine umfangreiche Expertise erfordert. Insbesondere muss versucht werden, die hohe Sterblichkeit der beatmeten Patienten zu senken [7]. Hier ist es entscheidend, dass Patienten in Krankenhäusern behandelt werden, die umfangreiche Erfahrungen mit der Beatmung haben. Patienten sollten entsprechend auch früh in diese Krankenhäuser verlegt werden, was, wie die vorliegenden Zahlen zeigen, nicht immer der Fall war (Abb. 5). Das Lungenversagen verläuft in verschiedenen Phasen [10] und insbesondere die ersten Tage sind hochvulnerabel. Hier bedarf es einer 24/7-Expertise in der Beatmungsmedizin mit einer entsprechend gut ausgestatteten pflegerischen und ärztlichen Besetzung. Schäden, die zu Beginn des durch COVID-19 ausgelösten Atemnotsyndrom (ARDS) gesetzt werden, können im weiteren Krankheitsverlauf oft nur schwer wieder behoben werden. Daher ist die Behandlung bzw. gegebenenfalls früheste mögliche Verlegung in Krankenhäuser mit entsprechender Ausstattung und Expertise notwendig.

Eine Konzentration der Fälle auf gut dafür ausgestattete Krankenhäuser ist auch bei der Durchführung von Studien, z. B. zur Wirksamkeit vorhandener Medikamente bei COVID-19 förderlich. So haben Großbritannien mit der RECOVERY-Studie zum Steroid Dexamethason [6] ebenso wie andere internationale Konsortien wie die REMAP-CAP-Studiengruppe deutlich gemacht, wie wichtig der Einschluss der Patienten in randomisiert-kontrollierte Studien ist. Strenge Studienbedingungen können jedoch häufig nur an großen und erfahrenen Krankenhäusern mit entsprechender Personalausstattung vorgehalten werden.

Auf Basis der Erfahrungen der ersten Pandemiewelle sollte daher für die zukünftige Versorgung ein klares Stufenkonzept entwickelt und konsequent umgesetzt werden. Einige Bundesländer, wie Hessen und Berlin, haben während der ersten Pandemiewelle ein solches Konzept aufgestellt. Die durch die Umsetzung der Konzepte zu erwartenden positiven Effekte waren auf der vorliegenden Datenbasis noch nicht analysierbar. Das Stufenkonzept in Hessen sieht die Behandlung von COVID-19-Patienten grundsätzlich nur in Universitätskliniken und Krankenhäuser mit Intensivmedizin, die die Möglichkeit des Einsatzes differenzierter Beatmungsverfahren haben, vor (Level-I-Krankenhäuser; [5]). Nur wenn deren Kapazitäten belegt sind, soll die Behandlung auch in anderen Krankenhäusern erfolgen. Auch in Berlin werden die Krankenhäuser, die über Intensivbetten verfügen, in 3 Level eingeteilt [9]. Am Level-I-Krankenhaus (Charité) erfolgt die Koordination der COVID-19-Patienten. Die Charité bildet mit 16 weiteren Level-II-Krankenhäusern das Netzwerk, in dem COVID-19-Patienten behandelt werden sollen. In den Level-III-Krankenhäusern in Berlin sollen zunächst keine COVID-19-Patienten versorgt werden, sondern die anderen Intensivpatienten. Wenn bei steigender Zahl der Neuinfektionen die vorgesehenen Kapazitäten für COVID-19-Patienten nicht mehr ausreichen, kommen entsprechend andere Umverteilungsmechanismen zum Tragen. Eine konsequente Umsetzung dieser Stufenkonzepte ist zum einen wichtig, um COVID-19-Patienten bestmöglich zu versorgen und um Patienten auf spezifische Krankenhäuser zu verteilen, die mit der Zeit mehr und mehr Erfahrung im Umgang mit COVID-19 sammeln; zum anderen aber auch, um die Versorgung von Patienten ohne COVID-19 klar gesteuert und separiert besser sicherstellen zu können.

Die vorliegende Studie weist einige Limitationen auf. So konnten in den Analysen zur Verteilung der Patienten nur AOK-Fälle betrachtet werden. Rund ein Drittel der deutschen Bevölkerung ist bei der AOK versichert. Die AOK-Fallzahlanteile auf Krankenhausebene können jedoch variieren. Für die Beatmungserfahrung konnten nur die Beatmungsstunden der AOK-Fälle einbezogen werden. Daher könnte sich auf Basis von bundesweiten Daten eine in Einzelfällen andere Quartilseinteilung der Krankenhäuser ergeben. Des Weiteren erfolgen die Analysen nur auf Ebene des Institutionskennzeichens (IK) des Krankenhauses. Jedoch kann ein IK mehrere Krankenhausstandorte umfassen, was zu einer Unterschätzung der Anzahl der Krankenhäuser führt und gegebenenfalls zu einer Überschätzung der Beatmungserfahrung, da sich die Beatmungsstunden auf mehr als ein Krankenhaus verteilen würden. Die Parameter Betten und Beatmungsstunden sind nur Hilfsgrößen für die Vorerfahrung eines Krankenhauses. Detaillierte Informationen, wie das Vorhandensein differenzierter Beatmungsverfahren oder einer ärztlichen Präsenz 24/7, liegen nicht vor. Des Weiteren können auf der ausgewerteten Datenbasis keine Aussagen über die Ergebnisqualität der Versorgung getroffen werden.

## Schlussfolgerungen

Im Rahmen einer zweiten Welle der Pandemie ist es notwendig, sowohl die Versorgungsstrukturen von COVID-19-Fällen durch klar definierte und zentral gesteuerte Stufenkonzepte zu verbessern als auch die notwendige Versorgung von Patienten ohne COVID-19 weiterhin aufrechtzuerhalten. Demenentsprechend wird für die Zukunft die Umsetzung umfassender Stufenkonzepte mit stärkerer Konzentration gefordert, wie sie beispielsweise in Hessen oder Berlin für die Versorgung von COVID-19-Patienten bereits vorliegen. Diese sollten bundesweit ohne Beachtung von Kreis- und Landesgrenzen umgesetzt werden. In Abhängigkeit der jeweiligen Auslastungsgrade der stationären Kapazitäten sollten darin unterschiedliche Stufen für die stationäre Versorgung vorgesehen werden. Hier sind Fachgesellschaften und die gesundheitspolitischen Akteure auf Bundesebene aufgefordert, allgemeingültige Ausstattungsmerkmale und Qualitätskriterien für ein Beatmungs- bzw. intensivmedizinisches Zentrum zu definieren. In einem ersten Schritt sollte die Freihaltung von Versorgungskapazitäten auf die Krankenhäuser konzentriert werden, die eine hohe Expertise in der Versorgung komplexer Krankenhausfälle aufweisen. Eine Ausdehnung der Freihaltung von Versorgungskapazitäten sollte in Abhängigkeit von realen Versorgungsbedarfen anhand von Daten über die Gesamtauslastung der Krankenhausversorgung erfolgen. Die Erfahrungen mit der COVID-19-Pandemie haben gezeigt, dass die Krankenhäuser innerhalb weniger Tage reaktionsfähig waren bzw. zusätzliche Kapazitäten freimachen konnten.

Damit ein solches Stufenkonzept umgesetzt werden kann, wird zum einen Transparenz über Ausstattungsmerkmale und Vorerfahrung der Krankenhäuser benötigt. Zum anderen muss ein stetiges Monitoring der verfügbaren stationären Behandlungskapazitäten insgesamt erfolgen. Zwar gibt es dies mit dem Register der Deutschen Interdisziplinären Vereinigung für Intensiv- und Notfallmedizin (DIVI) bereits für Intensivbetten, für die bestmögliche Patientensteuerung ist es allerdings in ausreichender Differenzierung nach allen Leistungsbereichen erforderlich. Darüber hinaus ist sicherzustellen, dass jedes Krankenhaus in der Lage ist, potenzielle Infektionsfälle zu erkennen, kurzfristig zu isolieren und entsprechend in ein zur längeren Versorgung geeignetes Krankenhaus zu verlegen.

## Supplementary Information





## Footnotes

1 Die Gruppe der Patienten, die in beiden Krankenhäusern beatmet worden sind, wird noch einmal aufgeteilt in Patienten, die nur 24 h im ersten Krankenhaus beatmet worden sind und in Patienten, die mehr als 24 h im ersten Krankenhaus beatmet worden sind.
