# Supplementary material for: Structure of hospital care for COVID-19 patients up to July 2020 in Germany
Source: Med Klin Intensivmed Notfmed. 2021 Jan 26;116(5):431–9. [Article in German] doi: 10.1007/s00063-021-00776-6 (PMC7837335; doi:10.1007/s00063-021-00776-6)
Supplement: Supplementary file 1 [file 63_2021_776_MOESM1_ESM.pdf]

Electronic Supplementary Material

Tabelle S1: Verteilung der Covid-19-Fälle auf Krankenhäuser nach verschiedenen Kriterien

						Verlegungsart					Fallcharakteristika			
**Fälle**	**Unterteilung**	**N**	**Anteil**	**N (KH)**	**Anteil**	**Keine**	**Weg-verl.**	**Zu-verl.**	**Zu-Weg-verl.**	**Divers**	**Alter**	**Männlich**	**Beatmungs-dauer in Tagen (Durchschnitt)**	**Verweil-dauer in Tagen (Durchschnitt)**
**Gesamt**														
Alle Fälle	Gesamt	17.094	100%	1.082	100%	79%	8%	8%	1%	3%	67,4	52%	2,0	12,9
Mit Beatmung	Gesamt	2.813	100%	674	100%	56%	18%	17%	4%	6%	67,4	67%	12,3	23,1
Ohne Beatmung	Gesamt	14.281	100%	1.059	100%	84%	6%	7%	0%	3%	67,4	49%	0,0	10,9
**Nach Quartilen auf Basis der AOK-Covid-19-Fallzahl**														
Alle Fälle	1. Quartil	521	3%	280	26%	51%	18%	20%	2%	9%	67,9	54%	1,8	12,4
Alle Fälle	2. Quartil	1.571	9%	273	25%	70%	12%	12%	1%	4%	66,6	52%	1,9	13,3
Alle Fälle	3. Quartil	3.487	20%	267	25%	78%	8%	9%	1%	4%	66,9	51%	2,1	13,6
Alle Fälle	4. Quartil	11.515	67%	262	24%	82%	7%	7%	1%	3%	67,6	53%	2,0	12,7
**Nach Quartilen auf Basis der AOK-Covid-19-Fälle mit Beatmung**														
Mit Beatmung	1. Quartil	216	8%	216	32%	57%	21%	10%	4%	8%	67,0	67%	11,1	20,5
Mit Beatmung	2. Quartil	246	9%	123	18%	55%	21%	15%	3%	6%	68,6	68%	11,2	22,3
Mit Beatmung	3. Quartil	710	25%	186	28%	58%	20%	14%	3%	5%	68,0	67%	12,8	24,1
Mit Beatmung	4. Quartil	1.641	58%	149	22%	54%	16%	19%	4%	7%	67,1	67%	12,5	23,1
**Nach Betten**														
Alle Fälle	1 bis 200 Betten	2.842	17%	353	33%	68%	9%	16%	1%	6%	70,2	50%	1,4	12,9
Alle Fälle	201 bis 500 Betten	6.703	39%	458	42%	82%	10%	5%	1%	3%	68,4	52%	1,7	12,1
Alle Fälle	Mehr als 500 Betten	7.549	44%	271	25%	81%	7%	8%	1%	3%	65,5	54%	2,6	13,6
Mit Beatmung	1 bis 200 Betten	389	14%	147	22%	41%	19%	24%	4%	11%	69,5	67%	10,3	22,3
Mit Beatmung	201 bis 500 Betten	992	35%	298	44%	60%	23%	9%	3%	5%	69,0	65%	11,5	21,8
Mit Beatmung	Mehr als 500 Betten	1.432	51%	229	34%	57%	13%	20%	4%	6%	65,8	68%	13,5	24,2
Ohne Beatmung	1 bis 200 Betten	2.453	17%	338	32%	72%	7%	15%	1%	5%	70,4	48%	0,0	11,4
Ohne Beatmung	201 bis 500 Betten	5.711	40%	452	43%	86%	8%	4%	0%	3%	68,3	49%	0,0	10,4
Ohne Beatmung	Mehr als 500 Betten	6.117	43%	269	25%	87%	5%	6%	0%	2%	65,4	50%	0,0	11,1
**Beatmungsstunden bei AOK-Fällen mit Pneumonie, Sepsis oder ARDS im Jahr 2019**														
Alle Fälle	1. Quartil (0 bis 2.797 Stunden)	1.669	10%	271	25%	64%	10%	18%	1%	7%	70,1	49%	0,9	12,2
Alle Fälle	2. Quartil (mehr als 2.797 bis 6.622 Stunden)	3.433	20%	270	25%	82%	11%	5%	0%	2%	68,7	51%	1,3	11,7
Alle Fälle	3. Quartil (mehr als 6.622 bis 14.052 Stunden)	4.838	28%	270	25%	84%	8%	5%	1%	3%	68,3	51%	1,8	12,4
Alle Fälle	4. Quartil (mehr als 14.052 Stunden)	7.154	42%	271	25%	78%	7%	10%	1%	3%	65,6	54%	2,8	13,9
Mit Beatmung	1. Quartil (0 bis 2.797 Stunden)	169	6%	80	12%	51%	32%	7%	2%	7%	70,0	70%	9,2	18,0
Mit Beatmung	2. Quartil (mehr als 2.797 bis 6.622 Stunden)	474	17%	164	24%	58%	30%	6%	2%	5%	69,5	65%	9,8	18,5
Mit Beatmung	3. Quartil (mehr als 6.622 bis 14.052 Stunden)	709	25%	192	28%	63%	17%	11%	3%	6%	68,0	63%	12,2	23,8
Mit Beatmung	4. Quartil (mehr als 14.052 Stunden)	1.461	52%	238	35%	52%	13%	24%	4%	7%	66,2	68%	13,6	24,8
Ohne Beatmung	1. Quartil (0 bis 2.797 Stunden)	1.500	11%	264	25%	66%	7%	19%	1%	7%	70,1	47%	0,0	11,6
Ohne Beatmung	2. Quartil (mehr als 2.797 bis 6.622 Stunden)	2.959	21%	266	25%	85%	8%	4%	0%	2%	68,5	49%	0,0	10,6
Ohne Beatmung	3. Quartil (mehr als 6.622 bis 14.052 Stunden)	4.129	29%	263	25%	87%	6%	4%	0%	2%	68,3	49%	0,0	10,5
Ohne Beatmung	4. Quartil (mehr als 14.052 Stunden)	5.693	40%	266	25%	85%	6%	6%	0%	2%	65,4	51%	0,0	11,2

Abbildung S1: Verteilung der Covid-19-Fälle (mit und ohne Beatmung) und Krankenhäuser nach der Beatmungserfahrung der Krankenhäuser im Jahr 2019 nach Aufnahmemonat, Anteil in %

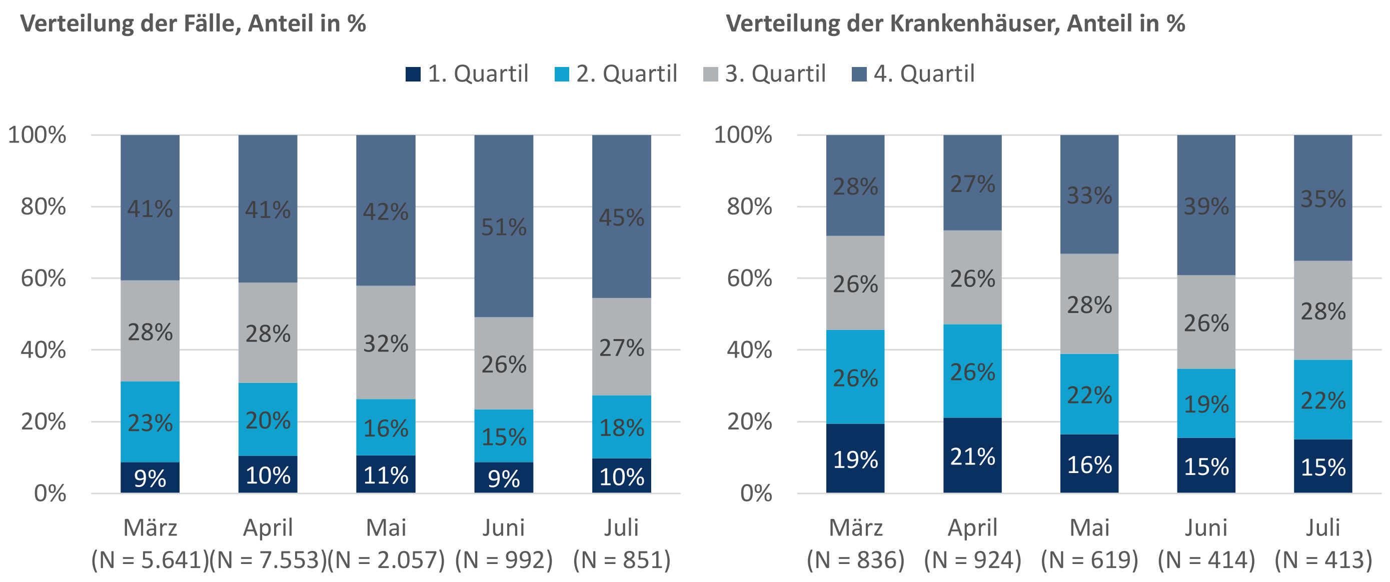

Anmerkung: Beatmungserfahrung definiert als Summe der Beatmungsstunden bei AOK-Fällen mit Pneumonie, Sepsis oder ARDS im Jahr 2019. Beatmungsstunden der AOK-Fälle: 1. Quartil – 0 bis 2.797 Stunden, 2. Quartil – mehr als 2.797 bis 6.622 Stunden, 3. Quartil – mehr als 6.622 bis 14.052 Stunden, 4. Quartil – mehr als 14.052 Stunden.

Abbildung S2: Krankenhäuser mit und ohne Beteiligung an der Covid-19-Versorgung, Anteil in %

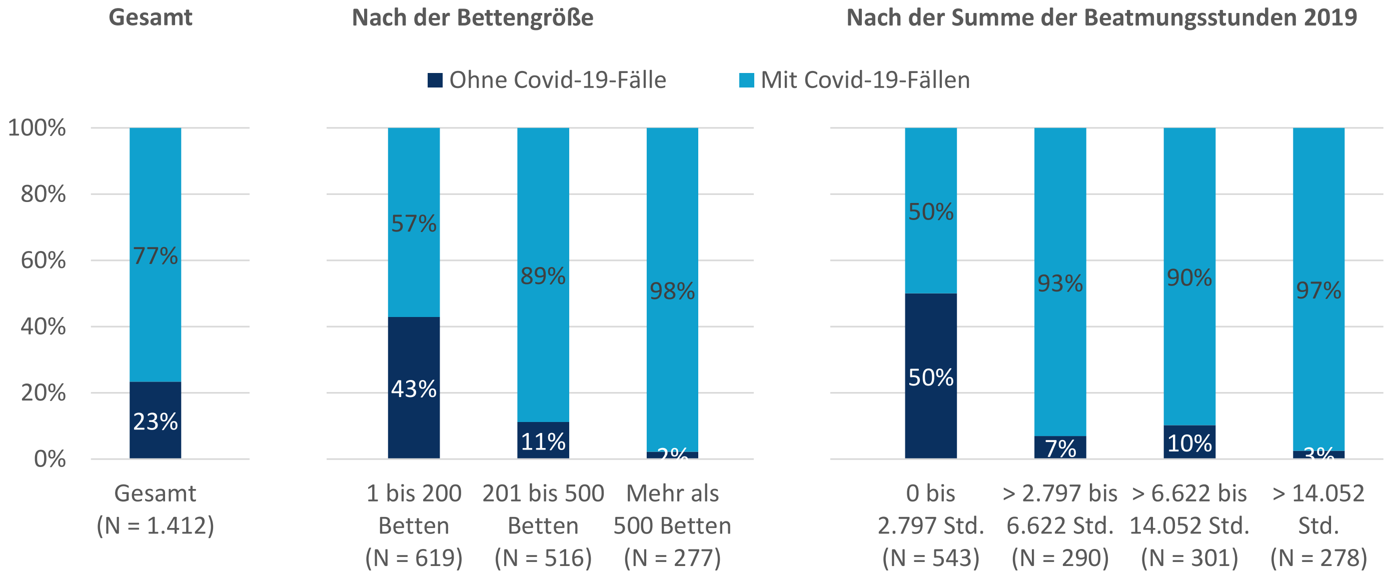

Anmerkung: Summe der Beatmungsstunden definiert aus den Beatmungsstunden bei AOK-Fällen mit Pneumonie, Sepsis oder ARDS im Jahr 2019. Beatmungsstunden ergeben sich aus der Quartilsverteilung der Krankenhäuser mit Covid-19-Behandlung.

Abbildung S3: Art der Verlegung, Anteil in %

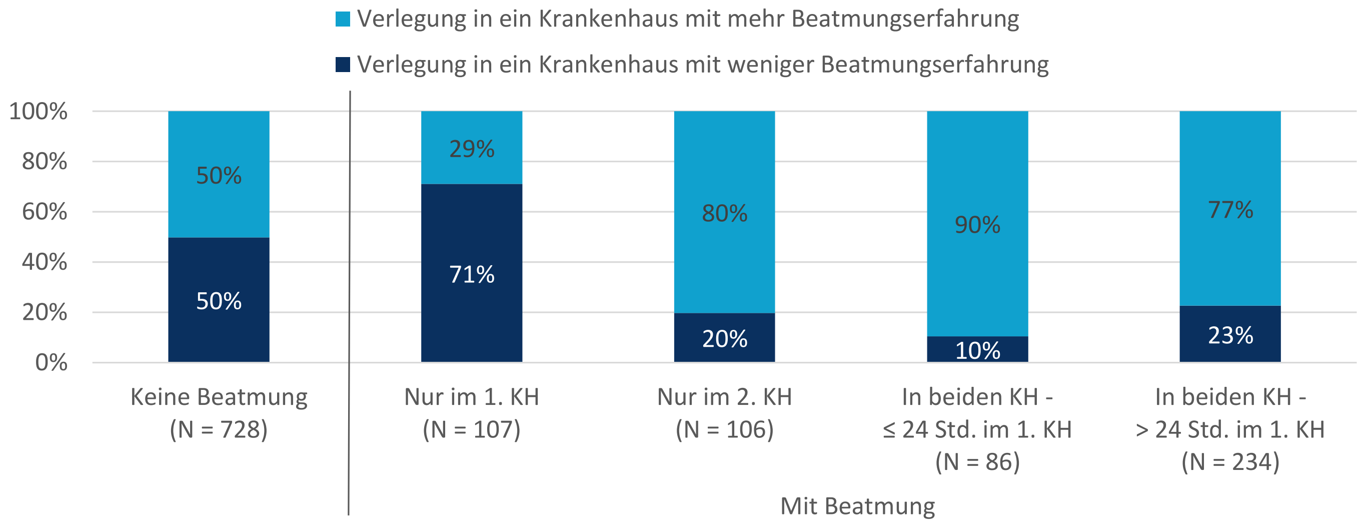

Anmerkung: Dargestellt werden Patienten mit einmaliger Verlegung. Ein Krankenhaus mit mehr Beatmungserfahrung ist definiert als ein Krankenhaus, welches mehr Beatmungsstunden bei Fällen mit Pneumonie, Sepsis und ARDS im Jahr 2019 aufweist, als das Krankenhaus, in welchem der Patienten zuerst behandelt wurde. KH – Krankenhaus.
